# Venous Thromboembolism in Patients with Glioblastoma: Molecular Mechanisms and Clinical Implications

**DOI:** 10.1055/s-0044-1789592

**Published:** 2024-08-21

**Authors:** Maaike Y. Kapteijn, Nina Bakker, Johan A. F. Koekkoek, Henri H. Versteeg, Jeroen T. Buijs

**Affiliations:** 1 Division of Thrombosis and Hemostasis, Department of Medicine, Einthoven Laboratory for Vascular and Regenerative Medicine, Leiden University Medical Center, Leiden, The Netherlands; 2 Department of Neurology, Leiden University Medical Center, Leiden, The Netherlands

**Keywords:** glioblastoma, venous thromboembolism, thromboprophylaxis, genomics

## Abstract

Patients with glioblastoma are among the cancer patients with the highest risk of developing venous thromboembolism (VTE). Long-term thromboprophylaxis is not generally prescribed because of the increased susceptibility of glioblastoma patients to intracranial hemorrhage. This review provides an overview of the current clinical standard for glioblastoma patients, as well as the molecular and genetic background which underlies the high incidence of VTE. The two main procoagulant proteins involved in glioblastoma-related VTE, podoplanin and tissue factor, are described, in addition to the genetic aberrations that can be linked to a hypercoagulable state in glioblastoma. Furthermore, possible novel biomarkers and future treatment strategies are discussed, along with the potential of sequencing approaches toward personalized risk prediction for VTE. A glioblastoma-specific VTE risk stratification model may help identifying those patients in which the increased risk of bleeding due to extended anticoagulation is outweighed by the decreased risk of VTE.

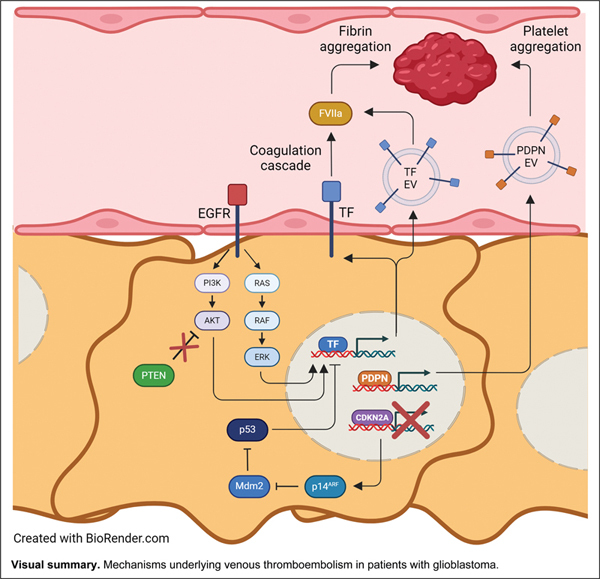

## Introduction

Glioblastoma, isocitrate dehydrogenase ( *IDH* )-wild-type, is the most frequent and the most aggressive type of primary brain cancer in adults, accounting for 48.6% of all malignant tumors within the central nervous system. 1 The overall annual incidence is 3.23 per 100,000 persons, which further increases with age and is higher in males compared to females. 1 2 The median expected survival time is 14.6 months despite extensive treatment, 3 with less than 5% of glioblastoma patients showing a survival rate of 5 years or more. 4 5

Glioblastoma is characterized by rapid proliferation, increased angiogenesis, hypoxia, and necrosis. 6 Vascular pathology, reflected by aberrant microvasculature and vascular leakage, induces a procoagulant state, which results in a high number of local (micro)thrombi within the tumor—histologically observed in 90% of all glioblastoma samples. 7 These in turn propagate tumoral hypoxia and necrosis, which together may contribute to systemic hypercoagulability. 6 Indeed, glioblastoma patients are at a high risk of developing venous thromboembolism (VTE), with an incidence of up to 10 to 30% per year. 8 9 This is one of the highest incidences among all cancer types. 10 The exact VTE risk may increase even further depending on patient-related, tumor-related, and therapy-related risk factors, such as age, tumor genetics, and surgery. 11 12

The complex pathogenesis of glioblastoma is also illustrated by a high degree of inter- and intratumoral heterogeneity. 13 Tumorigenesis is accompanied by a plethora of different mutations instead of a single-driver oncogene. This has great implications for glioblastoma prognosis and therapy, as treatment efficiency highly depends on tumoral gene expression. Moreover, the risk of developing VTE also relies on genetic signature and subsequent procoagulant gene and protein expression, the so-called coagulome. 14 Tissue factor (TF), the primary initiator of the coagulation cascade, and podoplanin, involved in platelet aggregation, are often upregulated in glioblastoma and assumed to significantly increase the risk of VTE. Prescription of thromboprophylaxis needs to be carefully considered due to the increased susceptibility of glioblastoma patients to intracranial hemorrhage. Altogether, these insights warrant a personalized benefit–risk evaluation in order to offer appropriate treatment for all glioblastoma patients.

This review provides an overview of the current clinical standard as well as the genetic and molecular background of glioblastoma-related VTE. Furthermore, potential novel biomarkers and future treatment strategies will be discussed in order to explore the prospects of personalized medicine for glioblastoma patients with an increased risk of VTE.

## Clinical Overview

### Glioblastoma Disease Course and Treatment

Patients with glioblastoma are often diagnosed from age 40 or higher. 15 Diagnosis requires histological and molecular characterization of tumor tissue, which also guides therapeutic management. Standard of care consists of surgery, pursuing maximum safe resection. 16 Surgical resection alone extends survival with 6 months approximately. 17 If resection is not feasible, a tumor biopsy is performed for diagnostic purposes. 18 In addition, glioblastoma patients are treated with a 6-week course of radiotherapy in combination with concomitant and adjuvant chemotherapy (temozolomide), also known as the Stupp protocol. 19 Elderly patients >70 years old have a worse prognosis and generally show lower tolerability of tumor-targeted treatment. For those patients, hypofractionated radiotherapy (3 weeks) with concomitant and adjuvant temozolomide and single modality treatment (monotherapy with either radiotherapy or temozolomide) are reasonable options. 20 Surgery combined with concomitant chemoradiotherapy increases overall survival to 14.6 months after glioblastoma diagnosis. 19 However, the response to temozolomide largely depends on promoter methylation of the gene *MGMT* , encoding O6-methylguanine-DNA methyltransferase (MGMT), since this DNA repair enzyme allows for reversal of temozolomide-induced DNA damage. 21 *MGMT* promoter methylation is observed in approximately 30 to 60% of all glioblastoma patients. 22

Treatment of glioblastoma recurrence is less well defined, but includes re-resection if possible. This improves survival, especially in case of a subtotal primary resection. 23 Additional treatment options are second-course chemotherapy, mostly lomustine, re-irradiation, and bevacizumab, a monoclonal antibody that inhibits angiogenesis by targeting the angiogenic protein vascular endothelial growth factor (VEGF). For patients with poor performance status, best supportive care is usually the most appropriate option. 24 Supportive care throughout the disease trajectory may consist of the glucocorticoid dexamethasone for tumor-related oedema, although this is associated with many side effects and may be related to poor treatment outcome. 25 26 For patients who develop epilepsy, antiseizure medication to reduce the risk of new seizures is standard of care. 27

### Venous Thromboembolism in Patients with Glioblastoma

The incidence of VTE in patients with glioblastoma is generally acknowledged within a range of 10 to 30% throughout the disease trajectory, 28 29 although reports vary from 7.5 to 39% depending on VTE definition, VTE detection method, and the use of thromboprophylaxis. 8 9 30 31 Most VTE events are observed within the postoperative period, but the risk remains higher over the course of the disease compared to other malignancies, with an incidence of 1.5 to 2.0% per month of survival. 32 A large retrospective study with malignant glioma patients ( *n* = 9,489) reported a 30% increased 2-year mortality rate in patients who developed VTE as compared to non-VTE patients. 33 However, this has not been confirmed by smaller studies with glioblastoma patients, possibly due to differences in VTE screening and management strategies. 34 35 36

The development of glioblastoma-related VTE depends on both general risk factors such as age, history of VTE, and comorbidity, and glioblastoma-specific risk factors such as peri-operative immobility, tumor recurrence, and subtotal resection. 28 Additional therapy-related factors comprise chemotherapy, bevacizumab, and dexamethasone. It is shown that chemotherapy results in a 3.4-fold increased VTE risk in cancer patients, 37 which may increase even further depending on the exact agent and protocol used. 38 Limited data are available for temozolomide and lomustine, the most commonly used chemotherapeutic agents in glioblastoma. Nevertheless, Yust-Katz et al demonstrated that the majority of VTE events developed after the start of adjuvant chemotherapy with temozolomide in a cohort of glioblastoma patients. 8 In line, our research group recently demonstrated increased TF-mediated procoagulant activity following treatment with temozolomide or lomustine in three well-established glioblastoma cell lines in vitro. 39 Furthermore, treatment with bevacizumab may increase the risk of pulmonary embolism in glioblastoma patients, as a trend toward significance was observed in bevacizumab-treated glioblastoma patients compared to glioblastoma patients who did not receive bevacizumab ( *p* = 0.07). 40 The risk of VTE may also be increased by high-dose glucocorticoid therapy such as dexamethasone, which directly affects the vascular endothelium. 41

### Intracranial Hemorrhage in Patients with Glioblastoma

In addition to VTE, tumor-related intracranial hemorrhage is also frequently observed in glioblastoma patients with reported incidences ranging from 2 to 12%, 9 42 either occurring as first manifestation or throughout the disease trajectory. 43 44 As was already described in 1982, the presence of a primary brain tumor by itself may cause spontaneous (nontraumatic) intracranial hemorrhage. 45 In glioblastoma, this is expected to be induced by increased expression of VEGF, which is involved in neovascularization and thereby contributes to vascular malformation and permeability. 46 47 The increased risk of major bleeding events as imposed by the tumor hampers the prescription of long-term thromboprophylaxis despite the significant risk of VTE in glioblastoma patients.

### Guidelines for Thromboprophylaxis

Primary thromboprophylaxis is recommended for ambulatory cancer patients on systemic anticancer therapy with a high risk of VTE, as assessed by VTE risk models. 48 49 The currently recommended VTE risk assessment score for chemotherapy-treated cancer patients with solid tumors (Khorana score) includes primary site of cancer (categorized into “very high risk” and “high risk”), body mass index, platelet count, leukocyte count, and hemoglobin level. 50 However, patients with brain tumors were underrepresented in the cohorts used to develop the Khorana score. As a result, brain cancer was insufficiently powered and not included as high-risk cancer type in this model. A subsequent retrospective cohort study with glioblastoma patients demonstrated that the Khorana score is lacking specificity for risk prediction of glioblastoma-related VTE. 8 This may not only be the consequence of underrepresentation, but also due to the fact that the included risk factors are not specifically relevant for glioblastoma. Ay et al proposed to include high-grade glioma as “very high risk” within the model. 51 Using this recommendation, an individual patient data meta-analysis by van Es et al reported an odds ratio (OR) of 3.5 (95% confidence interval [CI]: 0.89–14.0) for developing VTE in brain cancer patients with a high VTE risk (based on the Khorana score) compared to brain cancer patients with a Khorana score-based low-to-intermediate VTE risk. 52 However, these data were based on a small population and not statistically significant.

### Postoperative Thromboprophylaxis

Following the 2022 international clinical practice guidelines for the treatment and prophylaxis of VTE in patients with cancer, the use of low-molecular-weight heparin (LMWH) or unfractionated heparin is recommended postoperatively in cancer patients who are undergoing neurosurgery. The current standard for glioblastoma patients consists of postoperative treatment with LMWH for up to 10 days, which should be extended in case of prolonged immobilization. 53 Additionally, graduated compression stockings and/or intermittent pneumatic compression may be used perioperatively. 54

### Therapeutic Anticoagulation

For patients with brain tumors and established VTE, the use of LMWH or direct oral anticoagulants (DOACs) is recommended. 48 Several retrospective cohort studies with primary brain cancer patients have been performed to evaluate the risk of bleeding following therapeutic anticoagulation. A significantly increased incidence of intracranial hemorrhage was observed in a retrospective cohort study with glioblastoma patients receiving LMWH, heparin, or warfarin following a VTE event. 55 In line with this, retrospective data from high-grade glioma patients (of which 84.2% with glioblastoma) demonstrated a threefold increased risk of developing major intracranial hemorrhage in VTE patients receiving LMWH as compared to non-VTE patients (14.7 vs. 2.5%; hazard ratio [HR]: 3.37; 95% CI: 1.02–11.14; *p* = 0.036). 56 Another retrospective cohort study with high-grade glioma patients did not find an association between intracranial hemorrhage and the use of LMWH following VTE. 57 When comparing the use of LMWH and DOACs, both retrospective cohort studies specifically focusing on glioblastoma patients and studies with high-grade glioma patients demonstrate a lower incidence of intracranial hemorrhage in patients treated with DOACs compared to patients receiving LMWH. 58 59 60 This has led to increased preference for DOACs due to the possibility of oral administration. However, large cohort studies with glioblastoma patients are required to determine the exact influence of therapeutic anticoagulation on bleeding risk in this specific population.

### Long-Term Thromboprophylaxis

Long-term thromboprophylaxis for ambulatory cancer patients with LMWH is not generally prescribed because of the high bleeding risk, as recommended by the International Society on Thrombosis and Haemostasis. 49 The only trial on prolonged prophylaxis in high-grade glioma patients, the PRODIGE trial (dalteparin vs. placebo), noticed a trend towards reduced VTE in the first 6 months, with 9 out of 99 LMWH patients (9.1%) developing VTE compared with 13 out of 87 placebo patients (14.9%; HR: 0.51; 95% CI: 0.19–1.4; *p* = 0.29). Simultaneously, major bleeding occurred in 5 LMWH patients (5.1%) versus 1 placebo patient (1.1%; HR: 4.2; 95% CI: 0.48–36; *p* = 0.22). 61 However, this study was terminated prematurely due to expiration of study medication, resulting in incomplete sample size and consequently, low statistical power.

Two recent prospective trials on the use of DOACs did not include patients with primary brain cancer at all (the Cassini trial on rivaroxaban 62 ) or only a small number (the AVERT trial on apixaban, *n* = 24 63 ). Prospective data on long-term anticoagulation in larger cohorts of glioblastoma patients are unfortunately lacking. Due to potential advantages such as oral administration and relative safety, 60 prospective randomized clinical trials regarding the use of DOACs for long-term thromboprophylaxis in glioblastoma patients are warranted.

Altogether, the benefit–risk ratio of long-term thromboprophylaxis in glioblastoma patients is a delicate balance between the risk of developing life-threatening pulmonary embolism versus the risk of intracranial hemorrhagic events. Glioblastoma patients exhibit one of the highest risks of VTE in combination with increased susceptibility to intracranial hemorrhage, which warrants specialized prospective clinical trials and a glioblastoma-specific VTE risk assessment model. Risk stratification using novel procoagulant biomarkers may assist in identifying those patients with the highest risk of VTE, who may benefit from extended anticoagulation despite the increased risk of major bleeding.

## Molecular Background of Glioblastoma-Related VTE

Hypercoagulability directly depends on the activity of procoagulant proteins, which are involved in thrombus formation. There are several proteins with a physiological role in hemostasis that are upregulated within the glioblastoma tumor depending on tumor-specific features (e.g., genetic aberrations, hypoxia, vascularization). This has consequences within the tumor, resulting in local microthrombi, as well as systemically, since these procoagulant proteins are also secreted by the tumor or present on circulating tumor cells and tumor cell-derived extracellular vesicles (EVs), allowing for procoagulant activity at distant sites in the circulation. In the next paragraphs, two key proteins involved in glioblastoma-related VTE, podoplanin and TF, will be described in more detail.

### Podoplanin

Podoplanin is a transmembrane glycoprotein involved in platelet aggregation through the platelet-receptor CLEC-2. 64 In healthy tissue, podoplanin is strongly expressed in lymphatic endothelial cells, being a widely used marker for lymphatic development. 65 However, podoplanin is also frequently upregulated in several cancer types, such as skin cancer, lung cancer, germ cell cancer, and primary brain cancer, being involved in tumor progression, invasion, and metastasis. 66 Indeed, overexpression has been associated with epithelial-to-mesenchymal transition (EMT) and lymphangiogenesis, which correlates with poor survival in cancer patients. 65 Podoplanin is also expressed on circulating tumor cells and EVs in the blood, 67 68 which likely results in local platelet activation and, potentially, thrombus formation. 69 Furthermore, the release of pro-angiogenic factors such as VEGF from platelet granules contributes to tumor growth, angiogenesis, and increased hypercoagulability. 70

In glioblastoma, podoplanin overexpression strongly associates with intra-tumoral microthrombi and systemic VTE. Riedl et al described a sixfold increased risk of developing VTE in glioblastoma patients with high podoplanin expression levels compared to patients with low levels. 69 These patients also demonstrated low platelet counts and high D-dimer levels, presumably due to the consumption of platelets following podoplanin-induced platelet aggregation. Furthermore, a 2.6-fold increased mortality risk was observed in glioblastoma patients with high tumoral podoplanin expression.

Importantly, an association has been described between mutations within the gene *IDH1* and decreased expression of podoplanin (see below). 71 Since all glioblastoma tumors demonstrate *IDH* -wild-type expression following the most recent World Health Organization (WHO) classification of tumors of the central nervous system (2021), 72 glioblastoma patients inherently show high podoplanin levels and an increased VTE risk compared to patients with lower grade gliomas. This was confirmed in a recent cohort study with adult-type diffuse glioma (glioblastoma, *IDH* -wild-type vs. astrocytoma, *IDH* -mutant and oligodendroglioma, *IDH* -mutant and 1p/19q-codeleted), but no association was observed between the levels of circulating podoplanin and glioma subtype or cumulative VTE incidence. 73 Furthermore, Tawil et al reported increased platelet activation following injection of glioma-derived podoplanin-positive EVs in mice, but no significant increase in plasma D-dimer levels. Thus, although a link has been described between VTE and high tumoral podoplanin levels in glioblastoma patients (as compared to patients with low levels), more research is required to determine the role of circulating podoplanin in glioblastoma-related VTE.

### TF

TF is a transmembrane glycoprotein expressed on the surface of subendothelial cells. Upon vascular damage, TF may bind and activate its blood-borne ligand factor VII (FVII), resulting in the binary TF:FVIIa complex which activates FX into FXa. 74 This subsequently leads to thrombin generation, platelet activation, and conversion of fibrinogen into fibrin. Aggregation of platelets together with fibrin at the site of injury ultimately results in clot formation. Additionally, TF is involved in intracellular signaling through G-protein coupled protease-activated receptors (PARs), present on the cell membrane of platelets, (sub)endothelial cells, and cancer cells. 74 TF-mediated PAR2 signaling induces pro-angiogenic factors such as interleukin-8 (IL-8) and VEGF, 75 76 thereby contributing to several aspects of tumor progression such as migration, invasion, angiogenesis, and, potentially, hypercoagulability.

TF is upregulated in virtually all cancer types, resulting in TF expression within the tumor as well as in the circulation on circulating tumor cells and tumor-cell secreted EVs. 77 This contributes to the hypercoagulable state in cancer patients, increasing the risk of VTE, but also induces tumor progression through TF-mediated PAR signaling. 78 Because of being involved in both cancer and coagulation, TF is generally accepted as a protagonist that connects cancer and VTE. 79 80

In brain cancer, TF upregulation is frequently observed, being associated with grade of malignancy and vascular density. 81 82 Consequently, TF is highly expressed in glioblastoma. Furthermore, TF expression is induced following exposure to hypoxia, which is often seen in glioblastoma and was shown to enhance coagulation. 83 Indeed, in a small cohort of brain cancer patients ( *n* = 96), Thaler et al observed widespread TF expression in glioblastoma tumor tissue using immunohistochemical staining. 84 However, only 56 glioblastoma patients were included in this cohort (58.3%), and no correlation was found between TF expression and VTE. Nonetheless, as this study was clearly underpowered, an association between TF expression and risk of VTE in glioblastoma cannot be excluded.

The role of TF-positive EVs in cancer-associated thrombosis is still debated. Despite the fact that preclinical data emphasize the potential influence of circulating TF on VTE in cancer patients, 77 this could not readily be reproduced in clinical studies on ovarian cancer and non-small cell lung cancer. 85 86 Nevertheless, a direct link seems eminent in patients with pancreatic cancer. 87 88 89

Secretion of TF-bearing EVs by human glioblastoma cells was already described in 1984, being associated with platelet aggregation and thrombus formation. 90 In a small cohort of glioblastoma patients ( *n* = 61), TF-EV levels were found to be particularly high prior to surgery, with a further increase up to 7 months afterwards. 91 Increased TF-EV levels at 7 months after surgery were associated with subtotal tumor resection and radiological disease progression. Baseline mean TF-EV numbers were significantly higher in glioblastoma patients who developed VTE. In line with this, Unruh et al described a positive correlation between preoperative TF-EV activity and the risk of VTE in patients with *IDH* -wild-type glioma. 92 Furthermore, a recent study with adult-type diffuse glioma demonstrated a trend toward increased TF-EV activity in *IDH* -wild-type glioblastoma, with a significant association between highest TF-EV activity and both the fastest time to VTE and the highest cumulative VTE incidence. 73 However, in a study by Thaler et al, no significant association was found between TF-EV activity and development of VTE in brain cancer, 93 possibly due to differences in detection methods used as mentioned by Sartori et al. 91 Moreover, this cohort ( *n* = 119) consisted of high-grade glioma patients instead of specifically focusing on glioblastoma patients, and did not distinguish between *IDH* -wild-type and *IDH* -mutant glioma.

Since high TF-EV levels and activity can be directly linked to systemic hypercoagulability in selected cancer types including glioblastoma, these may be used as prognostic markers for the risk of glioblastoma-related VTE. Variation between studies may be explained by different detection methods assessing two different states of TF, being either encrypted or decrypted. 94 Decrypted TF induces coagulation by activating FX. In contrast, when surface-expressed TF is in an encrypted state, it induces intracellular signaling rather than exerting direct procoagulant activity. TF decryption is required to become fully active, which can be achieved by various stimuli ultimately resulting in increased exposure of phosphatidylserine (PS). In the presence of surface-expressed TF, the negatively charged PS stimulates formation of the procoagulant tenase and prothrombinase complexes, thus accelerating coagulation. 95 Additionally, TF decryption requires the formation of intracellular disulfide bonds between Cys ^186^ and Cys ^209^ as mediated by protein disulfide isomerase (PDI). 96 Since PDI is present on EV surfaces, this may induce formation of procoagulant TF-positive EVs. 97 However, the exact role of PDI-mediated TF decryption in cancer-associated thrombosis remains to be determined. 98 99

While decrypted TF directly contributes to VTE, cryptic TF induces tumor progression through TF-mediated PAR2 signaling, which indirectly may increase hypercoagulability through expression of VEGF and IL-8. 75 76 Thus, both forms of TF may increase the risk of VTE, especially in high-risk cancers such as glioblastoma. Since TF decryption allows for increased procoagulant activity on (circulating) tumor cells and TF-EVs, further inquiry about this process will contribute to our knowledge of the underlying molecular mechanisms of VTE in glioblastoma.

**Fig. 1:**
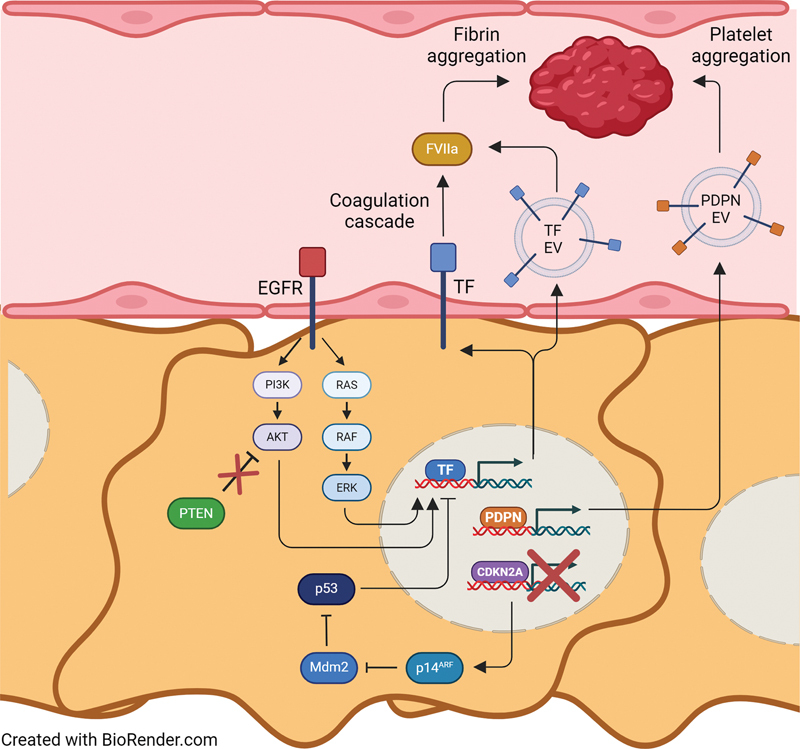
Proposed molecular and genetic mechanisms that underlie VTE in patients with glioblastoma. Upregulation of EGFR in glioblastoma cells results in increased signaling through PI3K–AKT and RAS–RAF–ERK, which both induce TF expression. Loss of PTEN prevents inhibition of AKT, which together with EGFR leads to increased expression of TF. Deletion of *CDKN2A* precludes expression of p14 ^ARF^ , which normally inhibits Mdm2, the negative regulator of p53. Consequently, Mdm2 represses p53 activity, resulting in TF upregulation. Altogether, this leads to increased expression of TF on the cell surface as well as on extracellular vesicles (EVs) in the circulation. Here, TF induces the coagulation cascade by activation of FVII into FVIIa, ultimately resulting in fibrin aggregation. On the other hand, increased expression of podoplanin in glioblastoma cells likely causes release of podoplanin-positive EVs, which induce platelet activation and aggregation through the platelet receptor CLEC-2. Collectively, in combination with TF-mediated fibrin aggregation, this results in clot formation, thus increasing the risk of VTE in patients with glioblastoma. EGFR, epidermal growth factor receptor; FVII, factor VII; TF, tissue factor; VTE, venous thromboembolism.

Both podoplanin and TF are considered to play a central role in glioblastoma-related VTE, as summarized in Fig. 1 . While high tumoral podoplanin expression has been shown to associate with VTE in glioblastoma, probably resulting in the secretion of podoplanin-bearing EVs, a positive correlation has also been described between the risk of glioblastoma-related VTE and both TF-EV levels and activity. Since systemic VTE in cancer presumably depends on increased hypercoagulability at distant sites from the tumor, as mediated by procoagulant EVs, TF may be a pivotal factor in glioblastoma-related VTE. This is in line with the most common genetic aberrations in glioblastoma, which are known to upregulate TF in several ways (see below). In addition, co-expression of TF and podoplanin within the glioblastoma tumor may have synergistic effects (see Fig. 1 ). In fact, in xenograft models it was shown that tumors expressing both TF and podoplanin demonstrated increased intravascular fibrin staining and vessel-occluding thrombi when compared to tumors expressing TF or podoplanin only. 68 Furthermore, tumoral expression of procoagulant proteins in glioblastoma may be highly heterogeneous, representing a mosaic of different glioblastoma subtypes which requires further investigation, e.g., by single-cell RNA sequencing. Thus, the exact influence of TF and podoplanin may differ per glioblastoma patient, and a certain degree of cooperation is very likely.

## Genetic Background of Glioblastoma-Related VTE

Inter- and intratumoral heterogeneity is a crucial and intrinsic hallmark of glioblastoma. This is captured by the different glioblastoma subtypes defined by Verhaak et al, who described a molecular classification system based on genetic signature. 100 While originally including four subtypes (i.e. classical, mesenchymal, proneural, and neural), more recent categorization resulted in a total of three (exclusion of the neural subtype 101 ), comprising glioblastoma, *IDH* -wild-type only following the most recent WHO classification of tumors of the central nervous system (2021). 72 Classical glioblastoma harbors the most common genetic aberrations in glioblastoma (hence the name), such as amplification of the epidermal growth factor receptor ( *EGFR* ) and homozygous deletion of *CDKN2A/B* . At the same time, well-known mutations in *TP53* , *NF1* , and *PDGFRA* are relatively underrepresented. 100 The mesenchymal subtype is mostly characterized by deactivating mutations in the gene *NF1* . Furthermore, this subtype is known for its high expression of mesenchymal and astrocytic markers (e.g., *CD44* , *MET* ), which leads to de- and transdifferentiated tumors due to the high degree of EMT. 100 102 Finally, proneural glioblastoma shows distinct alterations in *PDGFRA* or *TP53.* Additionally, because of increased expression of some well-known proneural development genes and stem-cell markers such as *SOX2* and Notch signaling proteins, the proneural signature is associated with cellular development and proliferation. 102

This molecular classification has great implications for glioblastoma prognosis and therapy, as treatment efficiency highly depends on molecular subtype. In terms of survival, mesenchymal glioblastoma is associated with worse prognosis as compared to nonmesenchymal glioblastoma (i.e., classical or proneural). 101 The risk of developing VTE also relies on glioblastoma subtype and subsequent procoagulant gene and protein expression, the so-called coagulome. 14 Interestingly, the mesenchymal subtype shows the most procoagulant gene expression profile, 14 although this has not been validated by looking at actual VTE events in a cohort with glioblastoma patients. In a nested case–control study with 46 glioblastoma patients, of which 23 with and 23 without VTE, our research group used RNA-sequencing data to explore a potential link between molecular glioblastoma subtype and VTE. Here, proneural/neural glioblastoma was identified as a potential risk factor (OR: 3.05; 95% CI: 0.81–10.17; *p* = 0.19). However, these data were not statistically significant. 103 Below, the most common genetic aberrations in glioblastoma are discussed, as well as their potential link with a hypercoagulable state.

### IDH1

Isocitrate dehydrogenase 1 (IDH1) is an enzyme of the citric acid cycle that normally converts isocitrate into α-ketoglutarate. Mutations in the *IDH1* gene, of which R132H is observed most frequently, are highly prevalent in glioma. 104 *IDH1* -mutant gliomas, currently classified as astrocytomas, *IDH* -mutant, are less aggressive and more sensitive to chemotherapy compared to glioblastoma, *IDH* -wild-type, resulting in better overall survival. 105 *IDH1* mutations are also associated with a decreased risk of VTE due to additional conversion of α-ketoglutarate into D-2-hydroxyglutarate, an oncometabolite that inhibits platelet aggregation. 92 Moreover, *IDH1* R132H mutation causes hypermethylation of the gene promoters of both *F3* , encoding TF, and *Pdpn* , encoding podoplanin, thus directly decreasing expression of the main procoagulant proteins involved in glioblastoma-related VTE. 71 92 Nevertheless, following the most recent WHO classification, all glioblastomas are classified as *IDH* -wild-type. 72 This may explain the increased risk of VTE in glioblastoma patients compared to lower grade gliomas, but does not allow for VTE risk stratification within the glioblastoma population.

### EGFR

EGFR is a transmembrane tyrosine kinase receptor involved in cell proliferation, differentiation, and migration. *EGFR* overexpression contributes to tumor growth and EMT, being associated with invasion and metastasis. 106 The *EGFR* gene is one of the most frequently mutated genes in glioblastoma, but less common in lower grade gliomas. 107 108 It is estimated that up to 60% of glioblastoma tumors show *EGFR* upregulation, either caused by genomic amplification, rearrangement, and/or mutation. 109 This is especially the case in classical glioblastoma, which is characterized by *EGFR* amplification. 100

The most common *EGFR* mutation in glioblastoma is EGFRvIII, which is caused by deletion of exon 2–7 and only found in cancer cells. EGFRvIII shows low constitutive activity and ligand-independent signaling, mainly through RAS and mTOR, resulting in increased proliferation, migration, and invasion. 106 In 1994, EGFRvIII-expressing glioma cell lines were already reported to induce increased tumorigenesis in nude mice. 110 Glioblastoma cells expressing EGFRvIII demonstrate upregulation of TF as well as other procoagulant proteins, such as PAR1, PAR2, and FVII. 111 112 Thus, constitutive signaling by EGFRvIII promotes tumor progression and a procoagulant microenvironment, suggesting a role in glioblastoma-related VTE. In line, expression of *EGFR* and TF was found to correlate in tumor specimens of patients with classical glioblastoma, 14 indicating an increased risk of VTE in glioblastoma patients with this specific subtype. However, to the best of our knowledge, a direct correlation between *EGFR* mutations, specifically EGFRvIII, and VTE in patients with glioblastoma has not been observed.

Intriguingly, high levels of *EGFR* and EGFRvIII expression in both glioblastoma cell lines and patient-derived glioblastoma stem cells correlated with low podoplanin expression. 68 Thus, *EGFR* conversely regulates the two main procoagulant proteins in glioblastoma, podoplanin, and TF.

### PTEN

*PTEN* is a tumor suppressor involved in cell-cycle control through inhibition of the PI3K–AKT signaling pathway. Loss of *PTEN* expression is common in all glioblastoma subtypes, with 20 to 40% of glioblastoma tumors harboring inactivating *PTEN* mutations and roughly 80% demonstrating loss of *PTEN* expression, e.g., by *PTEN* promoter methylation. 113 114 *PTEN* inactivation leads to upregulation of RAS and mTOR signaling and subsequent overexpression of TF, similarly to activation of *EGFR* . 83 Indeed, glioma cells that do not express *PTEN* show increased coagulation compared to cells with wild-type *PTEN* expression, especially during hypoxia, which was confirmed by increased TF levels in cell-culture media. This is in line with another study by Rong et al, in which *EGFR* overexpression led to TF upregulation in a *PTEN* -null glioblastoma cell line, which could be rescued by *PTEN* restoration. 115 Here, TF expression was found to be controlled by *PTEN* -mediated transcriptional regulation. Moreover, in non-small cell lung cancer patients, combined presence of inactivating mutations in *TP53* and *PTEN* was previously found to increase TF mRNA expression and decrease survival. 116

In addition to TF, an inverse correlation has been described between *PTEN* expression and podoplanin levels in glioblastoma cell lines in vitro, an in vivo mouse model, and primary glioblastoma samples, 117 which could very well explain upregulation of podoplanin in glioblastoma.

As *PTEN* is often inactivated in glioblastoma patients, its ability to regulate podoplanin and TF might explain the increased risk of VTE, especially in combination with oncogenic expression of *EGFR* . However, a direct correlation between *PTEN* activity and VTE in glioblastoma patients has not been described yet.

### CDKN2A/B

In addition to genetic alterations in *EGFR* and *PTEN* , homozygous deletion of *CDKN2A* and *CDKN2B* is also frequently observed in glioblastoma patients. Both genes are involved in cell-cycle control by regulating pRB and p53 signaling. 118 *CDKN2A* encodes both p16 ^INK4a^ and p14 ^ARF^ by alternative splicing, which were found to be inactivated in 52 and 49% of glioblastoma tumors, respectively. 119 In classical glioblastoma, 94% of samples showed homozygous deletion of *CDKN2A* in co-occurrence with amplification of *EGFR* . 100 *CDKN2A* deletion may exert a stimulating effect on TF expression, as p14 ^ARF^ suppresses TF-induced procoagulant activity in glioblastoma cells by regulating tissue factor pathway inhibitor-2 (TFPI-2). 120 Furthermore, p14 ^ARF^ normally inhibits Mdm2, the negative regulator of p53. Disruption of p14 ^ARF^ activity therefore results in inactivation of p53, which may lead to TF upregulation in combination with *KRAS* mutations, as described in colorectal cancer, or *PTEN* mutations, as described in non-small cell lung cancer. 116 121 Thus, *CDKN2A* deletion may increase hypercoagulability in glioblastoma patients by downregulation of TP53. Co-occurrence of *EGFR* amplification and *CDKN2A* deletion in classical glioblastoma may therefore result in an increased risk of developing VTE.

Homozygous deletion of *CDKN2B* , encoding p15 ^INK4b^ , has been described in 47% of all glioblastoma patients. 119 Interestingly, *CDKN2B* mutations were associated with a significantly increased risk of cancer-associated thrombosis in patients with solid tumors, independent of cancer type. 122 In the same study, *CDKN2A* was also part of the top 10 of somatic mutations that associate with an increased VTE risk in cancer patients, although significance was lost after false discovery rate adjustment. Moreover, our group recently reported a link between *CDKN2A* deletion and VTE in a cohort of 324 glioblastoma patients. 123 A targeted DNA-sequencing approach demonstrated a 12-month adjusted cumulative incidence of VTE of 12.5% in glioblastoma patients with a *CDKN2A* deletion, compared to 5.4% in glioblastoma patients with wild-type *CDKN2A* expression. This resulted in an HR of 2.53 (95% CI: 1.12–5.73, *p* = 0.026). Thus, frequently observed homozygous deletion of *CDKN2A/B* in glioblastoma patients may significantly increase the risk of VTE.

In the same study, we used the cBioPortal for Cancer Genomics to study a potential link between *CDKN2A* deletion and mRNA expression levels of podoplanin and TF. Based on the Glioblastoma Multiforme dataset of the PanCancer Atlas (TCGA), we found an inverse correlation between *CDKN2A* expression and podoplanin mRNA levels ( *p* = 0.009). 123 This is the only report on the relation between *CDKN2A/B* alterations and expression levels of podoplanin so far. A similar effect was observed for TF mRNA expression, although not statistically significant ( *p* = 0.058).

Altogether, there are several genetic aberrations that may affect the procoagulant genetic signature in glioblastoma patients, as summarized in Table 1 and Fig. 1 . Moreover, the aforementioned signaling pathways are all interconnected, thereby amplifying hypercoagulability. That is, *EGFR* amplification and *PTEN* deletion both induce RAS signaling, which is frequently overactivated in cancer and by itself induces a plethora of procoagulant effects, such as upregulation of pro-angiogenic VEGF or TF itself. 83 124 Furthermore, *TP53* mutations are often observed in proneural glioblastoma, and are known to promote TF expression in combination with inactivating alterations in either *PTEN* or *CDKN2A* . Intra-tumoral heterogeneity may impact the local tumor microenvironment even further, resulting in hypercoagulable niches within the tumor that affect the procoagulant systemic state. Thus, the combination of procoagulant mutations within the glioblastoma tumor may result in a patient-specific genetic risk profile for VTE, which needs to be fully addressed in order to identify glioblastoma patients with the highest risk of VTE. In this regard, sequencing approaches may be of great value to develop personalized treatment strategies for glioblastoma-related VTE.

**Table 1:** Common genetic aberrations in glioblastoma and their potential mechanistic impact on VTE

Gene	Type of alteration	Procoagulant effects on glioblastoma cell lines in vitro	Procoagulant effects in primary glioblastoma samples	Procoagulant effects in cohort studies with high-grade glioma patients
*EGFR*	Amplification, *EGFRvIII*	• Upregulation of procoagulant proteins such as TF, PAR1, PAR2, and FVII in *EGFRvIII* -expressing glioblastoma cell lines U-373 115 116 and U-87 116 • Decreased expression of podoplanin in glioblastoma cell lines U-373 and U-87 with wild-type *EGFR* , and in their counterparts expressing *EGFRvIII* 72	• Correlation with TF expression in tumor tissue of glioblastoma patients 14 119 • Decreased expression of podoplanin in patient-derived glioblastoma stem cells 72	
*PTEN*	Deletion	• Upregulation of TF through Akt and Ras signaling in combination with hypoxia, which could be decreased upon *PTEN* restoration in glioblastoma cell lines LN229 and U-87 87 • *PTEN* restoration results in reduction of *EGFR* -mediated TF expression in glioblastoma cell line U-87 119 • Increased expression of podoplanin through Akt signaling in glioblastoma cell lines LN18, LN229, LN428, U-87, and U-373 121	• Increased expression of podoplanin through Akt signaling in primary glioblastoma samples 121	
*CDKN2A*	Deletion	• Induction of TF-mediated procoagulant activity by reduction of *TFPI-2* expression in glioblastoma cell line LN229 124	• Inverse correlation with podoplanin mRNA expression (and TF mRNA expression, although not significant) in the Glioblastoma Multiforme dataset retrieved from the cBioPortal for Cancer Genomics 127	• Increased 12-month cumulative incidence of VTE as compared to *CDKN2A* wild-type expression in patients with glioblastoma (12.5 vs. 5.4%, respectively; HR: 2.53, 95% CI: 1.12–5.73, *p* = 0.026) 127
*CDKN2B*	Deletion			• Increased risk of cancer-associated thrombosis independent of tumor type, and increased 12-month cumulative incidence of VTE in patients with high-grade glioma specifically 126

Abbreviations: CI, confidence interval; HR, hazard ratio; TF, tissue factor; VTE, venous thromboembolism.

## Future Directions

Due to the poor overall survival of glioblastoma patients and the high burden of glioblastoma-related VTE, novel biomarkers and treatment strategies are highly warranted. Furthermore, a glioblastoma-specific risk assessment model for VTE may significantly improve decision making regarding the use of thromboprophylaxis. In the final part of this review, an overview is given of the potential future directions to further develop a personalized approach for preventing VTE in patients with glioblastoma.

### Current Biomarkers for Glioblastoma-Related VTE

Several studies have attempted to identify biomarkers to optimize risk calculation for VTE in glioblastoma. Suggested biomarkers mainly include clinical parameters for VTE risk prediction in the general population. In a study with high-grade glioma patients ( *n* = 141), of which 68.1% with glioblastoma, three potential biomarkers were identified: low platelet count, elevated D-dimer, and high soluble P (sP)-selectin. 125 In addition, FVIII activity and leukocyte count both showed borderline significance. Exploratory risk assessment models including either low platelet count and elevated sP-selectin or low platelet count, high leukocyte count, and elevated D-dimer resulted in a VTE probability of 23.0 and 37.7%, respectively. Furthermore, high D-dimer plasma levels, elevated von Willebrand factor levels, and decreased clotting time were associated with increased hypercoagulability in glioblastoma patients as compared to patients with meningioma. 126 Another study described a 2.1-fold increased VTE risk in high-grade glioma patients, of which 85% with glioblastoma, with elevated FVIII activity. 127 A benefit of these coagulation markers is their current diagnostic use in the clinic, resulting in detection methods being widely available. However, these parameters are not glioblastoma-specific, and the implementation of TF and/or podoplanin may further increase the prediction value of potential risk stratification tools for VTE in glioblastoma patients.

Intriguingly, in the general cancer population, high instead of low platelet count has been identified as a risk predictor for VTE. 50 128 This may be due to the fact that glioblastoma patients exhibit increased expression levels of podoplanin, which induces platelet aggregation and, consequently, platelet consumption. 69 This finding underlines the tumor-specific biology in brain cancer patients and further warrants a VTE risk assessment model for this specific population.

### Podoplanin as a Biomarker and Therapeutic Target for Glioblastoma-Related VTE

Tumoral podoplanin expression may be a relevant biomarker for glioblastoma-related VTE, as the risk of VTE is increased in glioblastoma patients with high podoplanin levels as compared to patients with low levels. 69 This can be examined by immunohistochemical staining of podoplanin-positive tumor tissue following surgical resection, although specific antibodies for tumor-expressed podoplanin are required as podoplanin is also expressed in healthy tissue. 129 Interestingly, the podoplanin-specific antibody NZ-1 could be used to block podoplanin-mediated platelet aggregation in glioblastoma cells, which may be useful for the reduction of VTE. 130 Furthermore, combination of this antibody with chimeric antigen receptor-transduced T-cells resulted in T-cell recognition of podoplanin-positive glioblastoma cells, which inhibited growth of glioma xenografts in vivo. 131 Thus, podoplanin may be a promising therapeutic target for glioblastoma, but the implications in a clinical setting remain to be investigated.

Circulating podoplanin is assumed to contribute to glioblastoma-related VTE as well, 68 69 132 but this has not been demonstrated so far. Nevertheless, since levels of circulating podoplanin have been measured in glioblastoma patients, 73 and podoplanin-induced platelet activation likely plays a role in thrombogenesis, the use of circulating podoplanin as a prognostic biomarker for glioblastoma-related VTE may be promising.

### TF as a Biomarker and Therapeutic Target for Glioblastoma-Related VTE

TF-EVs may be used as biomarker for VTE in glioblastoma, as both TF-EV levels and activity have been associated with increased VTE incidence. 73 91 A randomized controlled trial with advanced cancer patients reported a VTE risk reduction of 80% in patients with high TF-EV levels using thromboprophylaxis compared to high TF-EV patients without prophylactic treatment, 133 thus demonstrating the value of TF-EVs as prognostic biomarker for VTE. However, no glioblastoma patients were included in this study. Nevertheless, some promising TF-targeting treatment strategies for glioblastoma have been described over the last years. Induced expression of TFPI-2, which inhibits TF-mediated coagulation, resulted in impaired tumor growth and vessel formation in human glioblastoma cells in vitro and in vivo. 134 In line with this, the TF-targeting antibody 10H10 was shown to reduce tumor cell invasion and vascular activation in a human xenograft glioblastoma model. 135 Finally, the tick-derived TF inhibitor Ixolaris was found to block TF-induced procoagulant activity by attenuating tenase complex assembly in glioblastoma cells in vitro. 136 Furthermore, in vivo glioblastoma tumor growth in Ixolaris-treated mice was inhibited as a result of *VEGF* downregulation and decreased tumor vascularization. Thus, TF-targeting treatment strategies in glioblastoma may affect both tumor progression and prothrombotic activity. To date, TF-directed therapies are increasingly studied in clinical trials aimed at a broad spectrum of cancer types and stages, 137 but clinical studies specifically focusing at TF-related treatment for glioblastoma and glioblastoma-related VTE are warranted.

### Personalized Treatment Using Sequencing Approaches

Tumor genomics needs to be considered when assessing the risk of VTE in glioblastoma patients, since tumor heterogeneity and hypercoagulability are largely dictated by the underlying genetic profile of the tumor. DNA sequencing may therefore be a promising approach to discover novel VTE biomarkers. In the largest study to date to associate tumor-specific genetic aberrations with VTE ( *n* = 11,695), Dunbar et al described a link between increased VTE risk in patients with solid tumors and tumor mutations in *STK11* , *CDKN2B* , *KEAP1* , *KRAS* , *CTNNB1* , and *MET* . 122 This cohort consisted for 4% of high-grade glioma patients. Specifically focusing on a cohort with 324 glioblastoma patients, our research group showed that tumoral *CDKN2A* deletion is associated with an increased risk of VTE using targeted DNA sequencing. 123 Furthermore, we used next-generation RNA sequencing to discover novel tumor–expressed genes and signaling pathways that associate with glioblastoma-related VTE. This nested case–control study consisting of 23 glioblastoma patients with VTE and 23 glioblastoma patients without VTE demonstrated a potential role for Sonic Hedgehog signaling, with classical Sonic Hedgehog target gene *GLI1* showing the highest overexpression. 103 Taken together, every glioblastoma patient exhibits its own procoagulant profile, which warrants personalized treatment to determine the benefit–risk ratio of thromboprophylaxis. A glioblastoma-specific VTE risk assessment model including tumor genomics is required to identify glioblastoma patients in which the risk of bleeding due to extended anticoagulation is outweighed by the decreased risk of VTE.

## Conclusion

Glioblastoma patients are among the cancer patients with the highest risk of developing VTE. Increased local hypercoagulability is caused by a combination of vascular pathology and hypoxia, which is fueled into a systemic procoagulant state due to a variety of genetic aberrations within the tumor that affect the expression of procoagulant proteins. Particularly, the role of TF and podoplanin has been increasingly linked to hypercoagulability and development of VTE over the last years. In terms of a glioblastoma-specific VTE risk stratification model, expression levels of procoagulant EVs as well as readily available genomic markers may add great value to decision making about the use of thromboprophylaxis for glioblastoma patients. Potentially in combination with RNA-sequencing methods, this will lead to personalized VTE risk prediction which is required to improve future treatment strategies for glioblastoma-related VTE.

## References

[1] Ostrom Q T Patil N Cioffi G Waite K Kruchko C Barnholtz-Sloan J S CBTRUS statistical report: primary brain and other central nervous system tumors diagnosed in the United States in 2013-2017 Neuro-oncol 2020 22(12, Suppl 2):iv1 iv96 33123732 10.1093/neuonc/noaa200 PMC7596247

[2] Grochans S Cybulska A M Simińska D Epidemiology of glioblastoma multiforme-literature review Cancers (Basel) 2022 14 10 2412 35626018 10.3390/cancers14102412 PMC9139611

[3] Koshy M Villano J L Dolecek T A Improved survival time trends for glioblastoma using the SEER 17 population-based registries J Neurooncol 2012 107 01 207 212 21984115 10.1007/s11060-011-0738-7 PMC4077033

[4] (UK) National Cancer Information Network Brain Tumour Group Brodbelt A Greenberg D Winters T Williams M Vernon S Collins V P Glioblastoma in England: 2007-2011 Eur J Cancer 2015 51 04 533 542 25661102 10.1016/j.ejca.2014.12.014

[5] Bjorland L S Fluge O Gilje B Mahesparan R Farbu E Treatment approach and survival from glioblastoma: results from a population-based retrospective cohort study from Western Norway BMJ Open 2021 11 03 e043208 10.1136/bmjopen-2020-043208 PMC7959220 33712524

[6] Rong Y Durden D L Van Meir E G Brat D J ‘Pseudopalisading’ necrosis in glioblastoma: a familiar morphologic feature that links vascular pathology, hypoxia, and angiogenesis J Neuropathol Exp Neurol 2006 65 06 529 539 16783163 10.1097/00005072-200606000-00001

[7] Tehrani M Friedman T M Olson J J Brat D J Intravascular thrombosis in central nervous system malignancies: a potential role in astrocytoma progression to glioblastoma Brain Pathol 2008 18 02 164 171 18093251 10.1111/j.1750-3639.2007.00108.x PMC2610479

[8] Yust-Katz S Mandel J J Wu J Venous thromboembolism (VTE) and glioblastoma J Neurooncol 2015 124 01 87 94 25985958 10.1007/s11060-015-1805-2

[9] Kaptein F HJ Stals M AM Kapteijn M Y Incidence and determinants of thrombotic and bleeding complications in patients with glioblastoma J Thromb Haemost 2022 20 07 1665 1673 35460331 10.1111/jth.15739 PMC9320838

[10] Horsted F West J Grainge M J Risk of venous thromboembolism in patients with cancer: a systematic review and meta-analysis PLoS Med 2012 9 07 e1001275 22859911 10.1371/journal.pmed.1001275 PMC3409130

[11] Riedl J Ay C Venous thromboembolism in brain tumors: risk factors, molecular mechanisms, and clinical challenges Semin Thromb Hemost 2019 45 04 334 341 31041803 10.1055/s-0039-1688493 PMC6548560

[12] Tawil N Mohammadnia A Rak J Oncogenes and cancer associated thrombosis: what can we learn from single cell genomics about risks and mechanisms? Front Med (Lausanne) 2023 10 1.252417E6 10.3389/fmed.2023.1252417 PMC10769496 38188342

[13] Eisenbarth D Wang Y A Glioblastoma heterogeneity at single cell resolution Oncogene 2023 42 27 2155 2165 37277603 10.1038/s41388-023-02738-y PMC10913075

[14] Magnus N Gerges N Jabado N Rak J Coagulation-related gene expression profile in glioblastoma is defined by molecular disease subtype J Thromb Haemost 2013 11 06 1197 1200 23582031 10.1111/jth.12242

[15] Li K Lu D Guo Y Trends and patterns of incidence of diffuse glioma in adults in the United States, 1973-2014 Cancer Med 2018 7 10 5281 5290 30175510 10.1002/cam4.1757 PMC6198197

[16] Fernandes C Costa A Osorio L Current standards of care in glioblastoma therapy Brisbane (AU) Codon Publications 2017 197 241 29251860

[17] Lacroix M Abi-Said D Fourney D R A multivariate analysis of 416 patients with glioblastoma multiforme: prognosis, extent of resection, and survival J Neurosurg 2001 95 02 190 198 10.3171/jns.2001.95.2.0190 11780887

[18] Young R M Jamshidi A Davis G Sherman J H Current trends in the surgical management and treatment of adult glioblastoma Ann Transl Med 2015 3 09 121 26207249 10.3978/j.issn.2305-5839.2015.05.10 PMC4481356

[19] European Organisation for Research and Treatment of Cancer Brain Tumor and Radiotherapy Groups National Cancer Institute of Canada Clinical Trials Group Stupp R Mason W P van den Bent M J Radiotherapy plus concomitant and adjuvant temozolomide for glioblastoma N Engl J Med 2005 352 10 987 996 15758009 10.1056/NEJMoa043330

[20] Trial Investigators Perry J R Laperriere N O'Callaghan C J Short-course radiation plus temozolomide in elderly patients with glioblastoma N Engl J Med 2017 376 11 1027 1037 28296618 10.1056/NEJMoa1611977

[21] Butler M Pongor L Su Y T MGMT status as a clinical biomarker in glioblastoma Trends Cancer 2020 6 05 380 391 32348734 10.1016/j.trecan.2020.02.010 PMC7315323

[22] Weller M Stupp R Reifenberger G MGMT promoter methylation in malignant gliomas: ready for personalized medicine? Nat Rev Neurol 2010 6 01 39 51 19997073 10.1038/nrneurol.2009.197

[23] Bloch O Han S J Cha S Impact of extent of resection for recurrent glioblastoma on overall survival: clinical article J Neurosurg 2012 117 06 1032 1038 23039151 10.3171/2012.9.JNS12504

[24] Koekkoek J AF van der Meer P B Pace A Palliative care and end-of-life care in adults with malignant brain tumors Neuro-oncol 2023 25 03 447 456 36271873 10.1093/neuonc/noac216 PMC10013651

[25] Cenciarini M Valentino M Belia S Dexamethasone in glioblastoma multiforme therapy: mechanisms and controversies Front Mol Neurosci 2019 12 65 30983966 10.3389/fnmol.2019.00065 PMC6449729

[26] Zhou L Shen Y Huang T The prognostic effect of dexamethasone on patients with glioblastoma: a systematic review and meta-analysis Front Pharmacol 2021 12 727707 34531751 10.3389/fphar.2021.727707 PMC8438116

[27] van der Meer P B Taphoorn M JB Koekkoek J AF Management of epilepsy in brain tumor patients Curr Opin Oncol 2022 34 06 685 690 35838207 10.1097/CCO.0000000000000876 PMC9594141

[28] Perry J R Thromboembolic disease in patients with high-grade glioma Neuro-oncol 2012 14 04 iv73 iv80 23095833 10.1093/neuonc/nos197 PMC3480243

[29] Jenkins E O Schiff D Mackman N Key N S Venous thromboembolism in malignant gliomas J Thromb Haemost 2010 8 02 221 227 19912518 10.1111/j.1538-7836.2009.03690.x PMC2834309

[30] Simanek R Vormittag R Hassler M Venous thromboembolism and survival in patients with high-grade glioma Neuro-oncol 2007 9 02 89 95 17327573 10.1215/15228517-2006-035 PMC1871666

[31] Edwin N C Elson P Ahluwalia M S Khorana A A Venous thromboembolism in patients with glioblastoma: Risk factors and prognostic importance J Clin Oncol 2015 33(15, suppl):e13027 e13027

[32] Marras L C Geerts W H Perry J R The risk of venous thromboembolism is increased throughout the course of malignant glioma: an evidence-based review Cancer 2000 89 03 640 646 10931464 10.1002/1097-0142(20000801)89:3<640::aid-cncr20>3.0.co;2-e

[33] Semrad T J O'Donnell R Wun T Epidemiology of venous thromboembolism in 9489 patients with malignant glioma J Neurosurg 2007 106 04 601 608 17432710 10.3171/jns.2007.106.4.601

[34] Eisele A Seystahl K Rushing E J Venous thromboembolic events in glioblastoma patients: an epidemiological study Eur J Neurol 2022 29 08 2386 2397 35545894 10.1111/ene.15404 PMC9543144

[35] Lim G Ho C Roldan Urgoti G Leugner D Easaw J Risk of venous thromboembolism in glioblastoma patients Cureus 2018 10 05 e2678 30050733 10.7759/cureus.2678 PMC6059517

[36] Cote D J Dawood H Y Smith T R Venous thromboembolism in patients with high-grade glioma Semin Thromb Hemost 2016 42 08 877 883 27574964 10.1055/s-0036-1592334

[37] Mulder F I Horváth-Puhó E van Es N Venous thromboembolism in cancer patients: a population-based cohort study Blood 2021 137 14 1959 1969 33171494 10.1182/blood.2020007338

[38] Khalil J Bensaid B Elkacemi H Venous thromboembolism in cancer patients: an underestimated major health problem World J Surg Oncol 2015 13 204 26092573 10.1186/s12957-015-0592-8 PMC4486121

[39] Kapteijn M Y Zwaan S Ter Linden E Temozolomide and lomustine induce tissue factor expression and procoagulant activity in glioblastoma cells in vitro Cancers (Basel) 2023 15 08 2347 37190275 10.3390/cancers15082347 PMC10137012

[40] Li X Huang R Xu Z Risk of adverse vascular events in newly diagnosed glioblastoma multiforme patients treated with bevacizumab: a systematic review and meta-analysis Sci Rep 2015 5 14698 26423913 10.1038/srep14698 PMC4589758

[41] Walsh D C Kakkar A K Thromboembolism in brain tumors Curr Opin Pulm Med 2001 7 05 326 331 11584184 10.1097/00063198-200109000-00013

[42] Pan E Tsai J S Mitchell S B Retrospective study of venous thromboembolic and intracerebral hemorrhagic events in glioblastoma patients Anticancer Res 2009 29 10 4309 4313 19846992

[43] Zoccarato M Nardetto L Basile A M Giometto B Zagonel V Lombardi G Seizures, edema, thrombosis, and hemorrhages: an update review on the medical management of gliomas Front Oncol 2021 11 617966 33828976 10.3389/fonc.2021.617966 PMC8019972

[44] Giustozzi M Proietti G Becattini C Roila F Agnelli G Mandalà M ICH in primary or metastatic brain cancer patients with or without anticoagulant treatment: a systematic review and meta-analysis Blood Adv 2022 6 16 4873 4883 35772127 10.1182/bloodadvances.2022008086 PMC9631668

[45] Wakai S Yamakawa K Manaka S Takakura K Spontaneous intracranial hemorrhage caused by brain tumor: its incidence and clinical significance Neurosurgery 1982 10 04 437 444 7099393 10.1227/00006123-198204000-00004

[46] Cao R Eriksson A Kubo H Alitalo K Cao Y Thyberg J Comparative evaluation of FGF-2-, VEGF-A-, and VEGF-C-induced angiogenesis, lymphangiogenesis, vascular fenestrations, and permeability Circ Res 2004 94 05 664 670 14739162 10.1161/01.RES.0000118600.91698.BB

[47] Cheng S Y Nagane M Huang H S Cavenee W K Intracerebral tumor-associated hemorrhage caused by overexpression of the vascular endothelial growth factor isoforms VEGF121 and VEGF165 but not VEGF189 Proc Natl Acad Sci U S A 1997 94 22 12081 12087 9342366 10.1073/pnas.94.22.12081 PMC23709

[48] International Initiative on Thrombosis and Cancer (ITAC) advisory panel Farge D Frere C Connors J M 2022 international clinical practice guidelines for the treatment and prophylaxis of venous thromboembolism in patients with cancer, including patients with COVID-19 Lancet Oncol 2022 23 07 e334 e347 35772465 10.1016/S1470-2045(22)00160-7 PMC9236567

[49] Wang T F Zwicker J I Ay C The use of direct oral anticoagulants for primary thromboprophylaxis in ambulatory cancer patients: Guidance from the SSC of the ISTH J Thromb Haemost 2019 17 10 1772 1778 31353841 10.1111/jth.14564 PMC6773470

[50] Khorana A A Kuderer N M Culakova E Lyman G H Francis C W Development and validation of a predictive model for chemotherapy-associated thrombosis Blood 2008 111 10 4902 4907 18216292 10.1182/blood-2007-10-116327 PMC2384124

[51] Ay C Dunkler D Marosi C Prediction of venous thromboembolism in cancer patients Blood 2010 116 24 5377 5382 20829374 10.1182/blood-2010-02-270116

[52] IPDMA Heparin Use in Cancer Patients Research Group van Es N Ventresca M Di Nisio M The Khorana score for prediction of venous thromboembolism in cancer patients: an individual patient data meta-analysis J Thromb Haemost 2020 18 08 1940 1951 32336010 10.1111/jth.14824

[53] Taillibert S Taillandier L Le Rhun E Venous thrombosis in patients with high-grade glioma Curr Opin Oncol 2015 27 06 516 521 26447877 10.1097/CCO.0000000000000226

[54] ESA VTE Guidelines Task Force Faraoni D Comes R F Geerts W Wiles M D Force E VGT European guidelines on perioperative venous thromboembolism prophylaxis: neurosurgery Eur J Anaesthesiol 2018 35 02 90 95 29112542 10.1097/EJA.0000000000000710

[55] Khoury M N Missios S Edwin N Intracranial hemorrhage in setting of glioblastoma with venous thromboembolism Neurooncol Pract 2016 3 02 87 96 31386010 10.1093/nop/npv028 PMC6668262

[56] Mantia C Uhlmann E J Puligandla M Weber G M Neuberg D Zwicker J I Predicting the higher rate of intracranial hemorrhage in glioma patients receiving therapeutic enoxaparin Blood 2017 129 25 3379 3385 28468796 10.1182/blood-2017-02-767285

[57] Jo J Donahue J Sarai G Petroni G Schiff D Management of venous thromboembolism in high-grade glioma: does low molecular weight heparin increase intracranial bleeding risk? Neuro-oncol 2022 24 03 455 464 34383073 10.1093/neuonc/noab198 PMC8917403

[58] Reed-Guy L Desai A S Phillips R E Risk of intracranial hemorrhage with direct oral anticoagulants vs low molecular weight heparin in glioblastoma: a retrospective cohort study Neuro-oncol 2022 24 12 2172 2179 35551405 10.1093/neuonc/noac125 PMC9713497

[59] Carney B J Uhlmann E J Puligandla M Intracranial hemorrhage with direct oral anticoagulants in patients with brain tumors J Thromb Haemost 2019 17 01 72 76 30450803 10.1111/jth.14336

[60] Swartz A W Drappatz J Safety of direct oral anticoagulants in central nervous system malignancies Oncologist 2021 26 05 427 432 33523555 10.1002/onco.13698 PMC8100560

[61] Perry J R Julian J A Laperriere N J PRODIGE: a randomized placebo-controlled trial of dalteparin low-molecular-weight heparin thromboprophylaxis in patients with newly diagnosed malignant glioma J Thromb Haemost 2010 8 09 1959 1965 20598077 10.1111/j.1538-7836.2010.03973.x

[62] CASSINI Investigators Khorana A A Soff G A Kakkar A K Rivaroxaban for thromboprophylaxis in high-risk ambulatory patients with cancer N Engl J Med 2019 380 08 720 728 30786186 10.1056/NEJMoa1814630

[63] AVERT Investigators Carrier M Abou-Nassar K Mallick R Apixaban to prevent venous thromboembolism in patients with cancer N Engl J Med 2019 380 08 711 719 30511879 10.1056/NEJMoa1814468

[64] Suzuki-Inoue K Osada M Ozaki Y Physiologic and pathophysiologic roles of interaction between C-type lectin-like receptor 2 and podoplanin: partners from in utero to adulthood J Thromb Haemost 2017 15 02 219 229 27960039 10.1111/jth.13590

[65] Tammela T Alitalo K Lymphangiogenesis: molecular mechanisms and future promise Cell 2010 140 04 460 476 20178740 10.1016/j.cell.2010.01.045

[66] Astarita J L Acton S E Turley S J Podoplanin: emerging functions in development, the immune system, and cancer Front Immunol 2012 3 283 22988448 10.3389/fimmu.2012.00283 PMC3439854

[67] Shirai T Inoue O Tamura S C-type lectin-like receptor 2 promotes hematogenous tumor metastasis and prothrombotic state in tumor-bearing mice J Thromb Haemost 2017 15 03 513 525 28028907 10.1111/jth.13604

[68] Tawil N Bassawon R Meehan B Glioblastoma cell populations with distinct oncogenic programs release podoplanin as procoagulant extracellular vesicles Blood Adv 2021 5 06 1682 1694 33720339 10.1182/bloodadvances.2020002998 PMC7993100

[69] Riedl J Preusser M Nazari P M Podoplanin expression in primary brain tumors induces platelet aggregation and increases risk of venous thromboembolism Blood 2017 129 13 1831 1839 28073783 10.1182/blood-2016-06-720714 PMC5823234

[70] Mir Seyed Nazari P Riedl J Pabinger I Ay C The role of podoplanin in cancer-associated thrombosis Thromb Res 2018 164 01 S34 S39 29703483 10.1016/j.thromres.2018.01.020

[71] Sun C Xiao L Zhao Y Wild-type IDH1 and mutant IDH1 opposingly regulate podoplanin expression in glioma Transl Oncol 2020 13 04 100758 32208352 10.1016/j.tranon.2020.100758 PMC7097522

[72] Louis D N Perry A Wesseling P The 2021 WHO Classification of Tumors of the Central Nervous System: a summary Neuro-oncol 2021 23 08 1231 1251 34185076 10.1093/neuonc/noab106 PMC8328013

[73] Burdett K B Unruh D Drumm M Determining venous thromboembolism risk in patients with adult-type diffuse glioma Blood 2023 141 11 1322 1336 36399711 10.1182/blood.2022017858 PMC10082363

[74] Versteeg H H Heemskerk J W Levi M Reitsma P H New fundamentals in hemostasis Physiol Rev 2013 93 01 327 358 23303912 10.1152/physrev.00016.2011

[75] Hjortoe G M Petersen L C Albrektsen T Tissue factor-factor VIIa-specific up-regulation of IL-8 expression in MDA-MB-231 cells is mediated by PAR-2 and results in increased cell migration Blood 2004 103 08 3029 3037 15070680 10.1182/blood-2003-10-3417 PMC2837482

[76] Liu Y Mueller B M Protease-activated receptor-2 regulates vascular endothelial growth factor expression in MDA-MB-231 cells via MAPK pathways Biochem Biophys Res Commun 2006 344 04 1263 1270 16650817 10.1016/j.bbrc.2006.04.005

[77] Rondon A MR Kroone C Kapteijn M Y Versteeg H H Buijs J T Role of tissue factor in tumor progression and cancer-associated thrombosis Semin Thromb Hemost 2019 45 04 396 412 31096312 10.1055/s-0039-1687895

[78] Kocatürk B Versteeg H H Tissue factor isoforms in cancer and coagulation: may the best isoform win Thromb Res 2012 129 01 S69 S75 22682138 10.1016/S0049-3848(12)70020-8

[79] Date K Hall J Greenman J Maraveyas A Madden L A Tumour and microparticle tissue factor expression and cancer thrombosis Thromb Res 2013 131 02 109 115 23237339 10.1016/j.thromres.2012.11.013

[80] Tesselaar M E Romijn F P Van Der Linden I K Prins F A Bertina R M Osanto S Microparticle-associated tissue factor activity: a link between cancer and thrombosis? J Thromb Haemost 2007 5 03 520 527 17166244 10.1111/j.1538-7836.2007.02369.x

[81] Hamada K Kuratsu J Saitoh Y Takeshima H Nishi T Ushio Y Expression of tissue factor correlates with grade of malignancy in human glioma Cancer 1996 77 09 1877 1883 8646688 10.1002/(SICI)1097-0142(19960501)77:9<1877::AID-CNCR18>3.0.CO;2-X

[82] Guan M Jin J Su B Liu W W Lu Y Tissue factor expression and angiogenesis in human glioma Clin Biochem 2002 35 04 321 325 12135696 10.1016/s0009-9120(02)00312-0

[83] Rong Y Post D E Pieper R O Durden D L Van Meir E G Brat D J PTEN and hypoxia regulate tissue factor expression and plasma coagulation by glioblastoma Cancer Res 2005 65 04 1406 1413 15735028 10.1158/0008-5472.CAN-04-3376

[84] Thaler J Preusser M Ay C Intratumoral tissue factor expression and risk of venous thromboembolism in brain tumor patients Thromb Res 2013 131 02 162 165 23084660 10.1016/j.thromres.2012.09.020

[85] Cohen J G Prendergast E Geddings J E Evaluation of venous thrombosis and tissue factor in epithelial ovarian cancer Gynecol Oncol 2017 146 01 146 152 28501328 10.1016/j.ygyno.2017.04.021

[86] Gezelius E Flou Kristensen A Bendahl P O Coagulation biomarkers and prediction of venous thromboembolism and survival in small cell lung cancer: a sub-study of RASTEN - a randomized trial with low molecular weight heparin PLoS One 2018 13 11 e0207387 30412630 10.1371/journal.pone.0207387 PMC6226210

[87] Khorana A A Francis C W Menzies K E Plasma tissue factor may be predictive of venous thromboembolism in pancreatic cancer J Thromb Haemost 2008 6 11 1983 1985 18795992 10.1111/j.1538-7836.2008.03156.x PMC2848502

[88] Zwicker J I Liebman H A Neuberg D Tumor-derived tissue factor-bearing microparticles are associated with venous thromboembolic events in malignancy Clin Cancer Res 2009 15 22 6830 6840 19861441 10.1158/1078-0432.CCR-09-0371 PMC2783253

[89] van Es N Hisada Y Di Nisio M Extracellular vesicles exposing tissue factor for the prediction of venous thromboembolism in patients with cancer: a prospective cohort study Thromb Res 2018 166 54 59 29656167 10.1016/j.thromres.2018.04.009

[90] Bastida E Ordinas A Escolar G Jamieson G A Tissue factor in microvesicles shed from U87MG human glioblastoma cells induces coagulation, platelet aggregation, and thrombogenesis Blood 1984 64 01 177 184 6733271

[91] Sartori M T Della Puppa A Ballin A Circulating microparticles of glial origin and tissue factor bearing in high-grade glioma: a potential prothrombotic role Thromb Haemost 2013 110 02 378 385 23803674 10.1160/TH12-12-0957

[92] Unruh D Schwarze S R Khoury L Mutant IDH1 and thrombosis in gliomas Acta Neuropathol 2016 132 06 917 930 27664011 10.1007/s00401-016-1620-7 PMC5640980

[93] Thaler J Ay C Mackman N Microparticle-associated tissue factor activity, venous thromboembolism and mortality in pancreatic, gastric, colorectal and brain cancer patients J Thromb Haemost 2012 10 07 1363 1370 22520016 10.1111/j.1538-7836.2012.04754.x

[94] Rao L V Kothari H Pendurthi U R Tissue factor: mechanisms of decryption Front Biosci (Elite Ed) 2012 4 04 1513 1527 22201972 10.2741/477 PMC3883586

[95] Kunzelmann-Marche C Satta N Toti F The influence exerted by a restricted phospholipid microenvironment on the expression of tissue factor activity at the cell plasma membrane surface Thromb Haemost 2000 83 02 282 289 10739387

[96] Ahamed J Versteeg H H Kerver M Disulfide isomerization switches tissue factor from coagulation to cell signaling Proc Natl Acad Sci U S A 2006 103 38 13932 13937 16959886 10.1073/pnas.0606411103 PMC1599891

[97] Rothmeier A S Marchese P Langer F Tissue factor prothrombotic activity is regulated by integrin-arf6 trafficking Arterioscler Thromb Vasc Biol 2017 37 07 1323 1331 28495929 10.1161/ATVBAHA.117.309315 PMC5501484

[98] Stopa J D Zwicker J I The intersection of protein disulfide isomerase and cancer associated thrombosis Thromb Res 2018 164 01 S130 S135 29703471 10.1016/j.thromres.2018.01.005 PMC5929485

[99] Koizume S Miyagi Y Tissue factor in cancer-associated thromboembolism: possible mechanisms and clinical applications Br J Cancer 2022 127 12 2099 2107 36097177 10.1038/s41416-022-01968-3 PMC9467428

[100] Cancer Genome Atlas Research Network Verhaak R G Hoadley K A Purdom E Integrated genomic analysis identifies clinically relevant subtypes of glioblastoma characterized by abnormalities in PDGFRA, IDH1, EGFR, and NF1 Cancer Cell 2010 17 01 98 110 20129251 10.1016/j.ccr.2009.12.020 PMC2818769

[101] Wang Q Hu B Hu X Tumor evolution of glioma-intrinsic gene expression subtypes associates with immunological changes in the microenvironment Cancer Cell 2017 32 01 42 5.6E7 28697342 10.1016/j.ccell.2017.06.003 PMC5599156

[102] Phillips H S Kharbanda S Chen R Molecular subclasses of high-grade glioma predict prognosis, delineate a pattern of disease progression, and resemble stages in neurogenesis Cancer Cell 2006 9 03 157 173 16530701 10.1016/j.ccr.2006.02.019

[103] Kapteijn M Y Lanting V R Kaptein F HJ RNA-sequencing to discover genes and signaling pathways associated with venous thromboembolism in glioblastoma patients: a case-control study Thromb Res 2023 232 27 34 37918288 10.1016/j.thromres.2023.10.018

[104] Han S Liu Y Cai S J IDH mutation in glioma: molecular mechanisms and potential therapeutic targets Br J Cancer 2020 122 11 1580 1589 32291392 10.1038/s41416-020-0814-x PMC7250901

[105] Houillier C Wang X Kaloshi G IDH1 or IDH2 mutations predict longer survival and response to temozolomide in low-grade gliomas Neurology 2010 75 17 1560 1566 20975057 10.1212/WNL.0b013e3181f96282

[106] Wee P Wang Z Epidermal growth factor receptor cell proliferation signaling pathways Cancers (Basel) 2017 9 05 52 28513565 10.3390/cancers9050052 PMC5447962

[107] Crespo I Vital A L Gonzalez-Tablas M Molecular and genomic alterations in glioblastoma multiforme Am J Pathol 2015 185 07 1820 1833 25976245 10.1016/j.ajpath.2015.02.023

[108] Ekstrand A J James C D Cavenee W K Seliger B Pettersson R F Collins V P Genes for epidermal growth factor receptor, transforming growth factor alpha, and epidermal growth factor and their expression in human gliomas in vivo Cancer Res 1991 51 08 2164 2172 2009534

[109] TCGA Research Network Brennan C W Verhaak R G McKenna A The somatic genomic landscape of glioblastoma Cell 2013 155 02 462 477 24120142 10.1016/j.cell.2013.09.034 PMC3910500

[110] Nishikawa R Ji X D Harmon R C A mutant epidermal growth factor receptor common in human glioma confers enhanced tumorigenicity Proc Natl Acad Sci U S A 1994 91 16 7727 7731 8052651 10.1073/pnas.91.16.7727 PMC44475

[111] Magnus N Garnier D Rak J Oncogenic epidermal growth factor receptor up-regulates multiple elements of the tissue factor signaling pathway in human glioma cells Blood 2010 116 05 815 818 20462964 10.1182/blood-2009-10-250639

[112] Milsom C C Yu J L Mackman N Tissue factor regulation by epidermal growth factor receptor and epithelial-to-mesenchymal transitions: effect on tumor initiation and angiogenesis Cancer Res 2008 68 24 10068 10076 19074872 10.1158/0008-5472.CAN-08-2067 PMC2834285

[113] Duerr E M Rollbrocker B Hayashi Y PTEN mutations in gliomas and glioneuronal tumors Oncogene 1998 16 17 2259 2264 9619835 10.1038/sj.onc.1201756

[114] Baeza N Weller M Yonekawa Y Kleihues P Ohgaki H PTEN methylation and expression in glioblastomas Acta Neuropathol 2003 106 05 479 485 12904991 10.1007/s00401-003-0748-4

[115] Rong Y Belozerov V E Tucker-Burden C Epidermal growth factor receptor and PTEN modulate tissue factor expression in glioblastoma through JunD/activator protein-1 transcriptional activity Cancer Res 2009 69 06 2540 2549 19276385 10.1158/0008-5472.CAN-08-1547 PMC2759716

[116] Regina S Valentin J B Lachot S Lemarié E Rollin J Gruel Y Increased tissue factor expression is associated with reduced survival in non-small cell lung cancer and with mutations of TP53 and PTEN Clin Chem 2009 55 10 1834 1842 19661141 10.1373/clinchem.2009.123695

[117] Peterziel H Müller J Danner A Expression of podoplanin in human astrocytic brain tumors is controlled by the PI3K-AKT-AP-1 signaling pathway and promoter methylation Neuro-oncol 2012 14 04 426 439 22394497 10.1093/neuonc/nos055 PMC3309862

[118] Huang L E Impact of *CDKN2A/B* homozygous deletion on the prognosis and biology of IDH-mutant glioma Biomedicines 2022 10 02 246 35203456 10.3390/biomedicines10020246 PMC8869746

[119] Cancer Genome Atlas Research Network Comprehensive genomic characterization defines human glioblastoma genes and core pathways Nature 2008 455 7216 1061 1068 18772890 10.1038/nature07385 PMC2671642

[120] Zerrouqi A Pyrzynska B Brat D J Van Meir E G P14ARF suppresses tumor-induced thrombosis by regulating the tissue factor pathway Cancer Res 2014 74 05 1371 1378 24398474 10.1158/0008-5472.CAN-13-1951 PMC3947444

[121] Rao B Gao Y Huang J Mutations of p53 and K-ras correlate TF expression in human colorectal carcinomas: TF downregulation as a marker of poor prognosis Int J Colorectal Dis 2011 26 05 593 601 21404058 10.1007/s00384-011-1164-1

[122] Dunbar A Bolton K L Devlin S M Genomic profiling identifies somatic mutations predicting thromboembolic risk in patients with solid tumors Blood 2021 137 15 2103 2113 33270827 10.1182/blood.2020007488 PMC8057259

[123] Kapteijn M Y Kaptein F HJ Stals M AM Targeted DNA sequencing to identify genetic aberrations in glioblastoma that underlie venous thromboembolism; a cohort study Thromb Res 2023 221 10 18 36435047 10.1016/j.thromres.2022.11.013

[124] Meadows K N Bryant P Pumiglia K Vascular endothelial growth factor induction of the angiogenic phenotype requires Ras activation J Biol Chem 2001 276 52 49289 49298 11682481 10.1074/jbc.M108069200

[125] Thaler J Ay C Kaider A Biomarkers predictive of venous thromboembolism in patients with newly diagnosed high-grade gliomas Neuro-oncol 2014 16 12 1645 1651 24987133 10.1093/neuonc/nou106 PMC4232082

[126] Navone S E Guarnaccia L Locatelli M Significance and prognostic value of the coagulation profile in patients with glioblastoma: implications for personalized therapy World Neurosurg 2019 121 e621 e629 30292037 10.1016/j.wneu.2018.09.177

[127] Streiff M B Ye X Kickler T S A prospective multicenter study of venous thromboembolism in patients with newly-diagnosed high-grade glioma: hazard rate and risk factors J Neurooncol 2015 124 02 299 305 26100546 10.1007/s11060-015-1840-z PMC4659392

[128] Simanek R Vormittag R Ay C High platelet count associated with venous thromboembolism in cancer patients: results from the Vienna Cancer and Thrombosis Study (CATS) J Thromb Haemost 2010 8 01 114 120 19889150 10.1111/j.1538-7836.2009.03680.x

[129] Shibahara J Kashima T Kikuchi Y Kunita A Fukayama M Podoplanin is expressed in subsets of tumors of the central nervous system Virchows Arch 2006 448 04 493 499 16411134 10.1007/s00428-005-0133-x

[130] Kato Y Kaneko M K Kuno A Inhibition of tumor cell-induced platelet aggregation using a novel anti-podoplanin antibody reacting with its platelet-aggregation-stimulating domain Biochem Biophys Res Commun 2006 349 04 1301 1307 16979138 10.1016/j.bbrc.2006.08.171

[131] Shiina S Ohno M Ohka F CAR T cells targeting podoplanin reduce orthotopic glioblastomas in mouse brains Cancer Immunol Res 2016 4 03 259 268 26822025 10.1158/2326-6066.CIR-15-0060

[132] Jo J Diaz M Horbinski C Epidemiology, biology, and management of venous thromboembolism in gliomas: an interdisciplinary review Neuro-oncol 2023 25 08 1381 1394 37100086 10.1093/neuonc/noad059 PMC10398809

[133] Zwicker J I Liebman H A Bauer K A Prediction and prevention of thromboembolic events with enoxaparin in cancer patients with elevated tissue factor-bearing microparticles: a randomized-controlled phase II trial (the Microtec study) Br J Haematol 2013 160 04 530 537 23240761 10.1111/bjh.12163 PMC3609903

[134] Yanamandra N Kondraganti S Gondi C S Recombinant adeno-associated virus (rAAV) expressing TFPI-2 inhibits invasion, angiogenesis and tumor growth in a human glioblastoma cell line Int J Cancer 2005 115 06 998 1005 15723303 10.1002/ijc.20965

[135] Harter P N Dützmann S Drott U Anti-tissue factor (TF9-10H10) treatment reduces tumor cell invasiveness in a novel migratory glioma model Neuropathology 2013 33 05 515 525 23384223 10.1111/neup.12018

[136] Carneiro-Lobo T C Konig S Machado D E Ixolaris, a tissue factor inhibitor, blocks primary tumor growth and angiogenesis in a glioblastoma model J Thromb Haemost 2009 7 11 1855 1864 19624457 10.1111/j.1538-7836.2009.03553.x PMC2896491

[137] Ahmadi S E Shabannezhad A Kahrizi A Tissue factor (coagulation factor III): a potential double-edge molecule to be targeted and re-targeted toward cancer Biomark Res 2023 11 01 60 37280670 10.1186/s40364-023-00504-6 PMC10242999

